# Cyclone vulnerability assessment in the coastal districts of Bangladesh

**DOI:** 10.1016/j.heliyon.2023.e23555

**Published:** 2023-12-14

**Authors:** Showmitra Kumar Sarkar, Rhyme Rubayet Rudra, Md. Mehedi Hasan Santo

**Affiliations:** Department of Urban and Regional Planning, Khulna University of Engineering & Technology, Khulna-9203, Bangladesh

**Keywords:** Disaster risk, Emergency response, GIS, Coastal communities, Disaster preparedness

## Abstract

This research aims to assess the vulnerability to cyclones in the coastal regions of Bangladesh, employing a comprehensive framework derived from the Intergovernmental Panel on Climate Change (IPCC, 2007). The study considers a total of eighteen factors, categorized into three critical dimensions: exposure, sensitivity, and adaptive capacity. These factors are crucial in understanding the potential impact of cyclones in the region. In order to develop a cyclone vulnerability map, Principal Component Analysis (PCA) was applied, primarily focusing on the dimensions of sensitivity and adaptive capacity. The findings of this analysis revealed that sensitivity and adaptive capacity components accounted for a significant percentage of variance in the data, explaining 90.00 % and 90.93 % of the variance, respectively. Despite the lack of details about data collection, the study identified specific factors contributing significantly to each dimension. Notably, proximity to the coastline emerged as a highly influential factor in determining cyclone exposure. The results of this research indicate that certain areas, such as Jessore, Khulna, Narail, Gopalgonj, and Bagerhat, exhibit low exposure to cyclones, whereas regions like Chandpur and Lakshmipur face a high level of exposure. Sensitivity was found to be high in most areas, with Noakhali, Lakshmipur, and Chandpur being the most sensitive regions. Adaptive capacity was observed to vary significantly, with low values near the sea, particularly in locations like Cox's Bazar, Shatkhira, Bagerhat, Noakhali, and Bhola, and high values in regions farther from the coast. Overall, vulnerability to cyclones was found to be very high in Noakhali, Lakshmipur, Chandpur, and Bhola, low in Jessore and Khulna, and moderate in Barisal, Narail, Gopalgonj, and Jhalokati. These findings are expected to provide valuable insights to inform decision-makers and authorities tasked with managing the consequences of cyclones in the region.

## 1 Introduction

Tropical cyclones are among the worst natural catastrophes, with persistent strong winds, storm surges, and heavy rainfall [1]. Tropical cyclones cause significant loss of life, massive damage to property and the environment, and disruptions in communication networks [2]. The coastal areas are the most vulnerable to the cyclone disasters. Tropical cyclones pose greater risks to coastal populations and the environment than any other type of natural calamity, culminating in the greatest number of fatalities [1,3,4]. From 1968 to 2010, the world had an average of 88 tropical storms each year [5]. The most concerning factor is that out of the 88 tropical storms 48 attained the intensity of a tropical cyclone (categories 1 and 2), while 21 attained the intensity of a significant tropical cyclone (categories 3, 4, and 5) [1,5]. About 90 tropical cyclones emerge each year somewhere in the globe, and many of them become significant catastrophes [6]. Between 1970 and 2010, there were around 637 significant tropical cyclones recorded throughout the globe [1,2,5,7]. Cyclones cause an annual average of $26 billion USD in damage around the globe and killed over 1.9 million people [2,8]. Future climate change scenarios will increase the rate of cyclones in the near future [5] though there are a lot of debate between researchers about the matter [2,9]. But in the coming years, tropical cyclone impacts are anticipated to increase [[10], [11], [12]]. As a result, a significant number of coastal residents, businesses, and habitats would be severely impacted. Particularly in the context of climate change, which is addressed by the Intergovernmental Panel on Climate Change (IPCC) framework, the coastal impact of cyclones is a major concern. Cyclones, also known as hurricanes or typhoons in various regions of the globe, are destructive and powerful weather phenomena that frequently make landfall along coastlines. The IPCC framework evaluates the littoral impact of cyclones by taking into account a number of factors, including sea level rise, intensity and frequency, precipitation, and erosion. All of these factors can be influenced by climate change, resulting in a catastrophic cyclone [13].

In 2007, the Fourth Assessment Report (AR4) was issued by the IPCC, which presented a comprehensive framework for evaluating the susceptibility of coastal areas to climate change and the associated rise in sea levels. The paradigm presented herein offers a methodical approach for assessing the prospective consequences of climate change on coastal regions. The following are the fundamental elements of the IPCC framework for evaluating the susceptibility of coastal areas in the Fourth Assessment Report (AR4).: exposure, sensitivity, adaptive capacity and vulnerability assessment [14]. The updated IPCC 2014 AR5 underlined the growing vulnerability of coastal regions to cyclones due to climate change, with a particular focus on rising sea levels and increased storm intensity. It stressed the need for comprehensive and sustainable strategies to reduce this vulnerability, including adaptation measures, the preservation of natural coastal defenses, and social equity considerations to protect vulnerable communities. This information continues to inform policies and actions aimed at mitigating the impacts of cyclones in coastal areas around the world [[13], [14], [15]]. Several studies have been performed over the coastal region of the world to detect coastal vulnerability of cyclone using IPCC framework. For example: Multi-criterion analysis of cyclone risk along the coast of Tamil Nadu, India [16], tropical cyclone assessment using both cyclone track data and metrological data in India [17], multidimensional model for cyclone vulnerability assessment of urban slum dwellers in India [18], cyclone vulnerability assessment using GIS and Remote sensing in Tamil Nadu, India [19], cyclone-induced coastal vulnerability, livelihood challenges and mitigation measures of Matla-Bidya interfluve area, Indian Sundarbans [20], mapping the impact of climate change on the vulnerability of community livelihoods in the riparian zone of the Gangatic Plain, India [21], coastal vulnerability assessment using Landsat and Cubesat in Karachi, Pakistan [22], climate change vulnerability and adaptation options for the coastal communities of Pakistan [23], household vulnerability to floods and cyclones in Khyber Pakhtunkhwa, Pakistan [24], district level cyclone vulnerability assessment in Pakistan using geospatial techniques [25], effects of cyclone Hudhud captured by a high altitude automatic weather station in northwestern Nepal [26], disaster risk reduction and management in Nepal [27], assessment of tropical cyclone damage on dry forests using multispectral remote sensing in Mexico [28], impact of tropical cyclone in China [29] etc.

The risk assessment depicts how a system is likely to be impacted in the future by integrating the potential function of a hazard, exposure, and vulnerability [30]. Taking the right precautions may lessen the damage caused by deadly tropical cyclones. A proper risk, adaptive capacity and vulnerability assessment system can provide sufficient information to reduce the impact of a cyclone [31]. In theory, vulnerability is described as the degree to which people, resources, and ecosystems are vulnerable to the effects of certain hazards, as measured by physical, social, economic, and environmental factors [32]. Remote sensing and GIS is an effective way to detect vulnerability of spatial tropical coastal cyclone [33]. Several geospatial mapping methodologies have been used to estimate spatial tropical cyclone susceptibility [[34], [35], [36], [37]]. Multi-criteria integrating mapping techniques [30,[38], [39], [40]] and Fuzzy Analytical Hierarchy Process (FAHP) [41] are the most common as well as appropriate approach to detect cyclone vulnerability [39]. To perform a vulnerability assessment, many theoretical frameworks, conceptual models, and assessment procedures are also available such as pressure and release model [42], hazards-of-place model of vulnerability [43], vulnerability/sustainability framework by [44] etc.

Bangladesh is one of the most over populated countries in the world facing a lot of problems like climate change [10,[45], [46], [47], [48], [49]], deforestation [50,51], unplanned urbanization [50,52], pollution [34,38,[53], [54], [55]], manmade and natural disasters [15,56]. Bangladesh's coastline region has a more diversified and dynamic physical landscape than is often recognized. Bangladesh's coastline area is neither homogenous nor static. The land is dynamic, as are the people of Bangladesh [57]. Jessore, Narail, Gopalganj, Shariatpur, Chandpur, Satkhira, Khulna, Bagerhat, Pirozpur, Jhalakati, Barguna, Barisal, Patuakhali, Bhola, Lakshmipur, Noakhali, Feni, Chittagong, and Cox's Bazar are among the 19 coastal districts that make up Bangladesh's Coastal Zone [58]. The length of the coastline is 710 km, and it is made up of the interface of many biological and economic systems. These systems include tidal flats and mangroves (the world's biggest mangrove forest is 6017 km squared), among other things [59]. Coastal Bangladesh presents a wide array of livelihood options encompassing coastal and marine fishing, aquaculture (such as prawn and crab cultivation), agriculture, and the gathering of forest resources. In addition to the aforementioned economic activities, the south-west coast region engages in several other economic endeavors, including as salt production, seafood processing (specifically dry-fish production), day labor, and tourism, primarily centered around the Sundarbans and Kuakata areas [60]. Coastal region of Bangladesh is facing different catastrophic disasters which indulge climate change, storm, sea level rise, cyclone, storm surge, coastal inundation, salinity intrusion and land erosion etc. Cyclone is one of the most common and dangerous natural disasters among them. Tropical cyclones arise often in the Bay of Bengal during the early summer (April to June) and the late rainy season (September to November) [61]. Causalities of cyclone is very high in the country which includes death of 3500 people and incurred around $1.67 billion US dollars by Cyclone Sidr in 2007 [30], more than half a million homes were devastated by Cyclone Aila in 2009, and 190 people lost their lives [62]. But the most devasting fatal tropical cyclones occurred in 1970 and 1991 when around 500,000 and 140,000 people were killed [39]. Due to unpredictable climate change cyclones are more likely to occur in the coastal regions of Bangladesh which will put a large number of people at risk in the near future.

Several works have been performed to detect the risk and vulnerability of coastal tropical cyclones in Bangladesh which includes assessment of tropical cyclone in coastal region risk by [2,30], vulnerability assessment using influencing physical and socioeconomic factors [63], future scenario modeling [64], adaptation, recovery, and preparation of coastal communities to tropical cyclone effects [65], developing a physical, social and mitigation capacity index using FAHP and geospatial approach [39], adaptive practices in the coastal region [66], mapping of climate vulnerability of the coastal region using principal component analysis [67] etc. However, none of these studies have measured the condition of influential parameters such as exposure, sensitivity, and adaptive capacity in order to create a vulnerability map aggregating all of the parameters for Bangladesh's entire coastal region. In this work, multiple components such as exposure, sensitivity, and adaptive capacity were assessed using PCA and a geospatial method for the whole coastal area of Bangladesh, taking into account diverse factors such as socioeconomic and physical. Finally, a vulnerability map was constructed with the influential components in mind. Principal Components Analysis (PCA) is a current data analysis method that is extensively used to portray the vulnerability profile in decision-making based on geographical maps [67,68]. Our hypothesis posits that there is a notable spatial disparity in the susceptibility to cyclones throughout the entirety of the coastal area of Bangladesh. This variation is primarily influenced by disparities in exposure, sensitivity, and adaptive capability. The specific objectives of the research are as follows: 1) to conduct a comprehensive investigation into the current state of influential factors contributing to cyclone vulnerability within the coastal region of Bangladesh; 2) to utilize PCA and geospatial techniques to assess the spatial distribution of cyclone vulnerability, with an emphasis on pinpointing areas of high vulnerability; 3) to identify and delineate the regions most severely impacted by cyclone vulnerability, enabling targeted adaptation strategies and aiding policymakers, aid organizations, and the affected communities in effectively addressing these challenges.

This research will contribute to identify vulnerable areas, inform adaptation strategies, advance geospatial analysis techniques, and provide insights for other regions facing similar challenges. By developing a vulnerability map that takes into account influential factors such as exposure, sensitivity, and adaptive capacity, this study can help policymakers and aid organizations prioritize their efforts in the most vulnerable areas, as well as assist communities in vulnerable areas cope with stressors. The implications of the research findings have substantial importance for Bangladesh, a nation characterized by a high susceptibility to cyclonic events. Policymakers and humanitarian organizations can effectively allocate their resources and interventions by identifying the areas that are most susceptible to cyclones. This strategic approach allows for prioritization in decreasing the risks associated with cyclones and facilitating community adaptation to the adverse effects of severe weather events. The findings of this study have the potential to be applied in the development of early warning systems, evacuation strategies, and measures for disaster preparedness. The research findings could also serve as valuable input for the planning and design of coastal infrastructure, including seawalls and storm shelters. Moreover, the research has the potential to be utilized for the purpose of identifying regions that require significant assistance in terms of livelihood diversification, climate change adaption, and disaster risk reduction. In its whole, this study possesses the capacity to yield a substantial impact in mitigating the hazards associated with cyclones and enhancing the adaptability of coastal populations in Bangladesh. Furthermore, the consequences of this research extend beyond Bangladesh, as they hold relevance for other countries and regions that face vulnerability to cyclones. For instance, the findings of this study could be utilized to formulate universally applicable approaches for evaluating the susceptibility of coastal regions to cyclones. The research findings could potentially contribute to the formulation and implementation of international cooperation and aid initiatives aimed at mitigating cyclone risks and facilitating adaption measures.

## 2 Materials and methods

### 2.1 Description of the study area

**Fig. 1:**
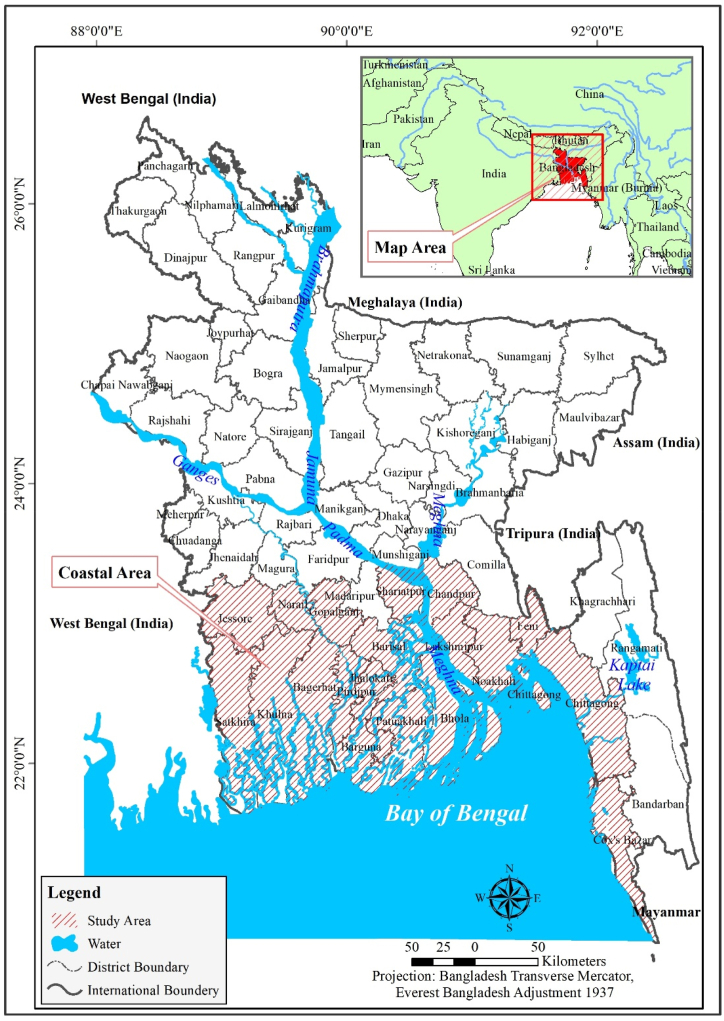
Study area.

Jessore, Narail, Gopalganj, Shariatpur, Chandpur, Satkhira, Khulna, Bagerhat, Pirozpur, Jhalakati, Barguna, Barisal, Patuakhali, Bhola, Lakshmipur, Noakhali, Feni, Chittagong, and Cox's Bazar are among the 19 coastal districts of Bangladesh [58] (Fig. 1). Based on geographical characteristics, the Bangladeshi coastline is divided into three zones: a. the eastern zone b. the central zone, c. the western zone. The Ganges tidal plain is a semi-active delta crisscrossed by numerous channels and streams in the northwestern part of India. The accretion and erosion processes are most active and continuous in the central region. The Meghna estuary is located in this region. The northeastern region is dominated by mountainous terrain that is more stable [69]. Bangladesh's coastal zone encompasses 47,201 km^2^, which is around 32% of the country. Approximately 35 million individuals, or 29 % of the population, inhabit in this zone [58].

### 2.2 Description of the data

Vulnerability to tropical cyclones has to be evaluated using different parameters. The geographical data utilized for assessing the criterion was compiled using geospatial methods. The total of eighteen indicators were categorized into three component classes (i.e., exposure, sensitivity, and adaptive capacity). In Table 1, we describe the data sets that formed the basis of our investigation. The timeline of the data was (2022–2023). All the data were selected based on previous studies [2,30]. In this study normalized value have been used but in order to show the distribution precisely we have used different parameter.

**Table 1:** Exposure, sensitivity and adaptive capacity components.

Component	Criteria	Min	Max	Mean	S.d.	Source
Exposure	Distance to Cyclone track (km)	0.00	56.41	8.51	8.26	Bangladesh Institute of Development Studies
	Distance to Coastline (km)	0.02	77.78	18.75	20.55	
Sensitivity	Elevation (m)	3.65	28.38	8.57	4.24	United States Geological Survey
	Slope	0.16	3.75	0.81	0.60	
	Population Density (sq. km)	107.57	48391.78	3128.23	7214.10	Bangladesh Bureau of Statistics
	Rural Population (%)	0.00	100.00	77.85	27.32	
	Female Population (%)	44.85	54.30	50.53	1.74	
	Population between 0 and 6 years old (%)	10.17	22.65	15.39	2.55	
	Population ages 65 and above (%)	1.66	7.41	5.17	1.25	
	Poverty headcount ratio (%)	0.01	64.37	32.37	16.49	
	Primary employment: Agriculture (%)	0.26	81.62	48.82	22.45	
Adaptive Capacity	Working-age Population (%)	0.50	0.74	0.59	0.05	
	Primary employment: Industry (%)	1.75	57.94	11.79	9.31	
	Literacy Rate (%)	25.27	82.25	55.34	11.48	
	Households with Electricity (%)	7.09	99.23	52.65	23.69	
	Distance from Coastal Vegetation (km)	0.00	96.53	11.43	21.03	Survey of Bangladesh
	Distance from Health Centre (km)	0.61	20.40	2.09	2.70	
	Distance from Major Road (km)	0.43	25.17	2.62	3.53	

### 2.3 Analytical method

**Fig. 2:**
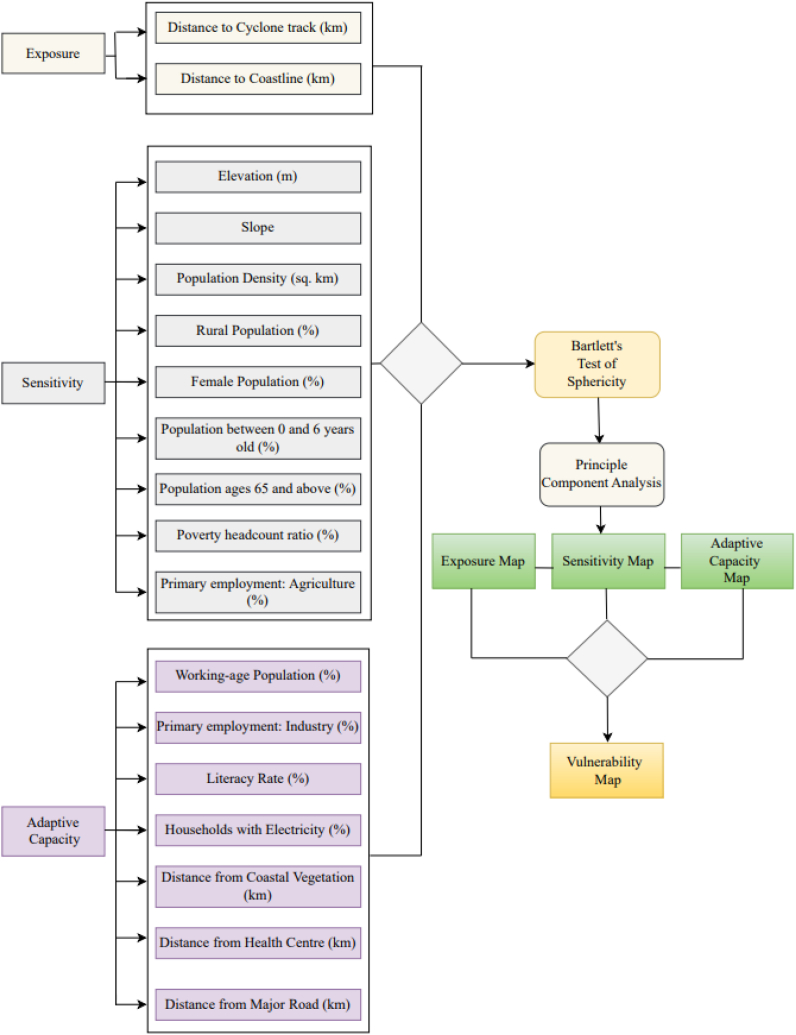
Methodological framework.

In this study vulnerability to natural disaster is used as an analytical framework. Fig. 2 shows the overall methodological framework. Exposure, sensitivity and adaptive capacity are used as function of expression for vulnerability. Under exposure component two criteria were selected such as distance to track in km and distance to coastline in km. Distance to Cyclone Track (km) variable is crucial as it indicates how close an area is to the cyclone's path, which directly influences the level of exposure and Proximity to the coastline is a key factor in understanding the vulnerability of an area to storm surges and other coastal impacts during a cyclone. Using the euclidian distance and natural breaks tools in GIS, these two maps were generated [39]. On the contrary, nine criteria were selected for sensitivity for example: elevation, slope, population density, rural population %, female population %, population between 0 and 6 years old (%), population ages 65 and above (%) etc. Lower elevations may be more susceptible to flooding during a cyclone, making this an important sensitivity indicator and areas with steep slopes might experience landslides or increased runoff, contributing to the vulnerability during cyclones [70]. These two maps were created using DEM data and slope tool. Demographic factors are very important to show the sensitivity for example: Rural areas may have different adaptive capacities and infrastructure compared to urban areas, gender-specific vulnerabilities may need to be considered during cyclones, age grouping is really important to see whether there are vulnerable age groups whom are less resilient to cyclone disasters [71,72]. These demographic factors were calculated using raster calculator. For adaptive capacity different infrastructural distance and demographic criteria were selected. Manmade structures such as health centers critical for emergency medical response and major road can affect the speed and efficiency of evacuation and relief efforts which are very important factors for adaptive capacity [73]. These criteria maps were created using euclidean distance and natural break classification [67]. Four different demographic factors were selected for adaptive capacity for example: working age population which is important to indicate the potential workforce available for recovery efforts, primary employment industries which is important for recovery capacities, literacy percentage which is crucial to know how many people are familiar with early warning signings, household with electricity percentage that can impact the ability to cope with and recover from cyclone-related disruptions [47,71,72]. Table 2 shows GIS and RS tools, source and resolution. According to the 2014 report by the IPCC, vulnerability results from the interaction of exposure, sensitivity, and adaptive capacity [74]. Vulnerability depends on the type, magnitude, and rate of climate change and variation to which a system is exposed, as well as its sensitivity and adaptability [75]. Exposure is considered as an external stressor that leads to susceptibility, while IPCC does not provide a definition [76]. Thus, we can define vulnerability as the sensitivity of a system, which is mitigated by its adaptability, and the first-order effect of exposure [70].

(1) Vulnerability = Exposure + Sensitivity - Adaptive Capacity

**Table 2:** GIS and RS tools, source and resolution.

Component	Criteria	Resolution	GIS Tools	Source
Exposure	Distance to Cyclone track (km)	30 m	Euclidean Distance < Natural Break Classification	Bangladesh Institute of Development Studies
	Distance to Coastline (km)	30 m	Euclidean Distance < Natural Break Classification	
Sensitivity	Elevation (m)	30 m	DEM Data < Natural Break Classification	United States Geological Survey
	Slope	30 m	Slope tool < Natural Break Classification	
	Population Density (sq. km)	30 m	Statistical Data < Natural Break Classification	Bangladesh Bureau of Statistics
	Rural Population (%)	30 m	Raster Calculation < Natural Break Classification	
	Female Population (%)	30 m	Raster Calculation < Natural Break Classification	
	Population between 0 and 6 years old (%)	30 m	Raster Calculation < Natural Break Classification	
	Population ages 65 and above (%)	30 m	Raster Calculation < Natural Break Classification	
	Poverty headcount ratio (%)	30 m	Raster Calculation < Natural Break Classification	
	Primary employment: Agriculture (%)	30 m	Raster Calculation < Natural Break Classification	
Adaptive Capacity	Working-age Population (%)	30 m	Raster Calculation < Natural Break Classification	
	Primary employment: Industry (%)	30 m	Raster Calculation < Natural Break Classification	
	Literacy Rate (%)	30 m	Raster Calculation < Natural Break Classification	
	Households with Electricity (%)	30 m	Raster Calculation < Natural Break Classification	
	Distance from Coastal Vegetation (km)	30 m	Euclidean Distance < Natural Break Classification	
	Distance from Health Centre (km)	30 m	Euclidean Distance < Natural Break Classification	Survey of Bangladesh
	Distance from Major Road (km)	30 m	Euclidean Distance < Natural Break Classification	

Cyclone exposure, sensitivity and adaptive capacity are determined by different independent variables. Sensitivity and adaptability factors are grouped in a way that makes their numbers go up as sensitivity or adaptability goes up. For instance, poverty headcount ratio and distance from health center have a huge impact to increase coastal vulnerability. The description of each independent factors and source of these factors are given in Table 1. ArcGIS is used for analysis and making the maps of each independent variables. The maps that are made are put into groups using the natural break classification method, which is found to be the best way to see the pattern of vulnerability in space in this study [77]. Spatial resolution of each raster cell is 30 × 30 m.

Multicollinearity is when a large number or all of the independent variables in a dataset are linked in a linear way. This makes it hard to figure out the relationship between each independent and dependent variable [78]. When one independent variable is substantially associated with another independent variable in a multiple regression equation, this phenomenon is known as multicollinearity. Inadequate control for multicollinearity reduces the validity of statistical findings [70]. PCA is a popular method of multivariate analysis that reduces a large number of potentially correlating variables to a smaller number of relatively independent ones [79]. In this study, multivariate analysis is used to eliminate multicollinearity in data.

Factor loadings of sensitivity and adaptive capacity were computed using Varimax rotation; these two factors exhibit a negative correlation with the factor score. Principal component analysis sample size was determined using the Kaiser-Meyer-Olkin (KMO) and Bartlett's Test of Sphericity [80]. The KMO value must be greater than or equal to 0.50 for the PCA to be considered statistically significant. In order to be considered significant, a component must account for more than a third of the variation in the data and meet the Kaiser Eigenvalue requirement (>1). To determine Cyclone vulnerability, researchers employed standardized measures of exposure, sensitivity, and adaptive capability. The information is normalized using Equation (2).

(2) $$x^{\prime }=\frac{x‐\mathrm{Min}\left(x\right)}{\mathrm{Max}\left(x\right)‐\mathrm{Min}\left(x\right)}$$

Where, $x^{\prime }$ = normalized data of a district; x = data of a district; Min(x) = the minimum value among the districts; Max(x) = the maximum value among the districts.

To normalize Narail's exposure, for example distance from coastline for each district was calculated. Then the highest and lowest distance of the districts situated to the coastlines were noted. After that, we used the min-max normalization method (Equation (2)) to get Narail's normalized exposure. The main benefit of min-max normalization is that it creates a range of values from 0 to 1, with 0 being the lowest possible value and 1 the greatest possible value.

After calculating the parameters such as the exposure, sensitivity and adaptive capacity the vulnerability of the coastal region was determined using Equation (1). Then the vulnerability map of the coastal region was classified into five groups using Jenks’ natural breaks classification method. Arc GIS 10.5 was used to create the maps of the vulnerability components, criteria and final vulnerability map. For statistical analysis IBM SPSS 25 was used. The normalization formula utilized for a particular sort of relationship is shown as Equation (2) in the paper. It is crucial to emphasize that the normalization process exhibits variability contingent upon whether the association between the variable and vulnerability (or its constituent elements) is positive or negative. The formula provided pertains only to variables in which an increase in value signifies a heightened level of vulnerability. In situations when a greater numerical value is associated with decreased vulnerability or a positive outcome, it would be necessary to modify the normalization algorithm accordingly. The formula described in Equation (2) is utilized for variables that exhibit a positive connection, where larger values suggest heightened susceptibility. In the case of variables exhibiting a negative connection, wherein higher values correspond to decreased vulnerability, it becomes necessary to adapt the formula in order to accurately depict higher values as advantageous or indicative of lower risk. This adjustment can require reversing the direction of the normalization process or adopting a different formula altogether, depending on the characteristics of the variable and its relationship to vulnerability.

## 3 Results

### 3.1 Spatial distribution of parameters

#### 3.1.1 Description of exposure parameters

**Fig. 3:**
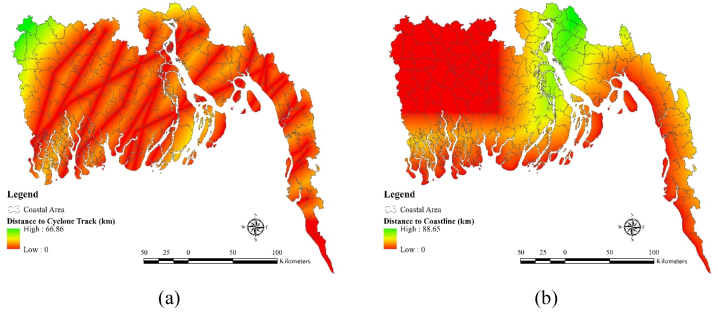
Exposure Factors: (a) Distance to coastline, (b) Distance to Cyclone track.

In this study, two independent factors such as i) distance to coastline, ii) distance to Cyclone track, were used as the exposure factors. Results show that north eastern part especially Feni, Noakhali and Chittagong district are quite far from the coastline compare to other regions which is around 88.65 km (Fig. 3(a)). Other than that, most of the regions are pretty close to the coastline. North western side of the region especially the part of the Shatkhira and Jessore are far from the cyclone center which is around 66.86 km (Fig. 3(b)).

#### 3.1.2 Description of the sensitivity parameters

**Fig. 4:**
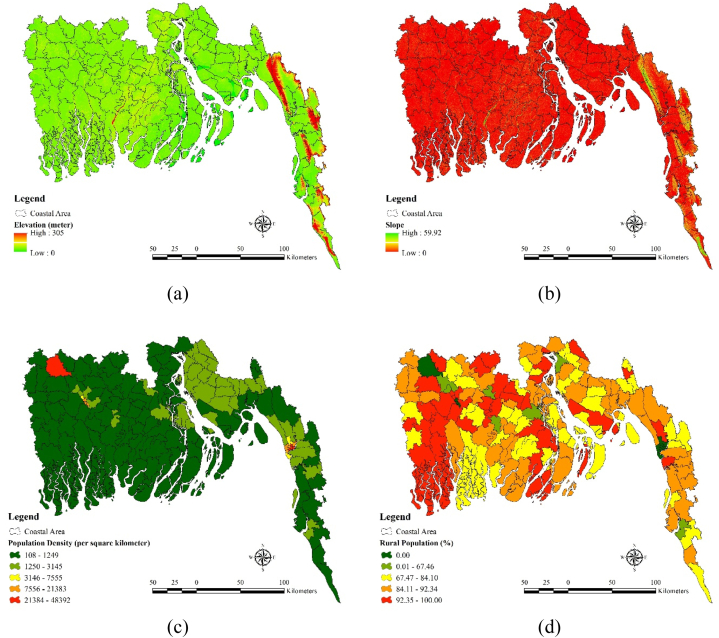

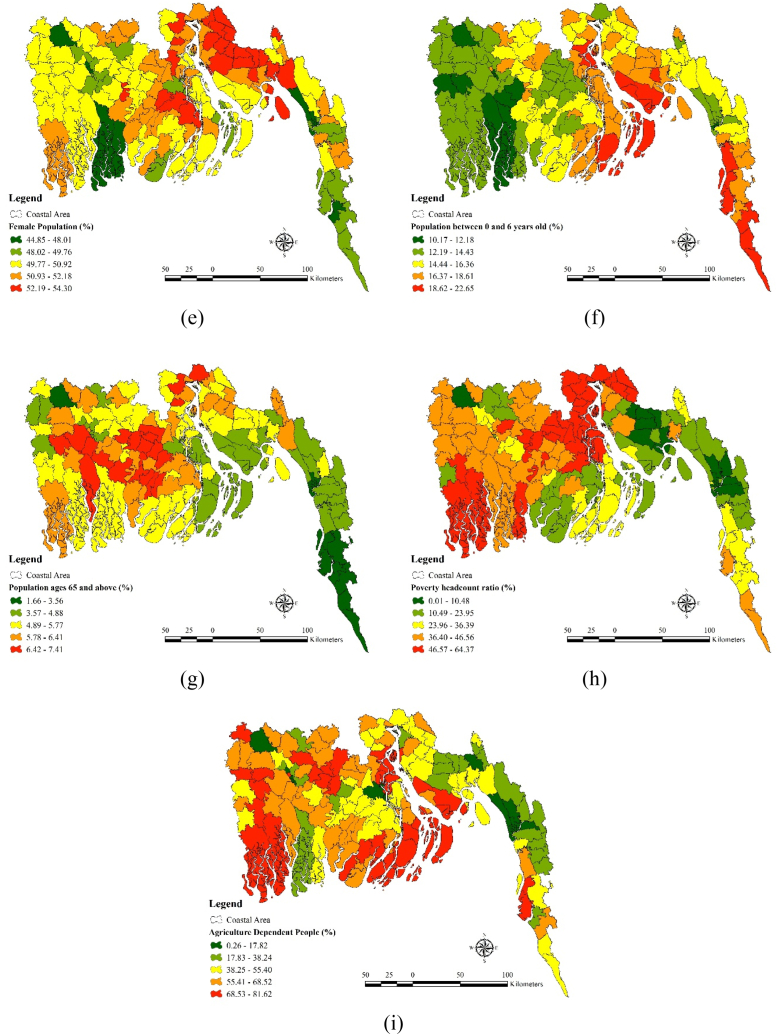
Sensitivity Factors: (a) Elevation, (b) Slope, (c) Population density, (d) Rural population, (e) Female population, (f) Population between 0 and 6 years old, (g) Population ages 65 and above, (h) Poverty head count ratio, (i) Agriculture dependent people.

Nine factors such as i) slope, ii) elevation, iii) population density iv) rural population v) female population vi) population between 0 and 6 years old (%) vii) population ages 65 and above (%) viii) poverty head count ratio (%) ix) agriculture dependent people were used as the variables for sensitivity analysis. Some hilly tracks situated in the south eastern region contain higher elevation (305) value (Fig. 4(a)) and higher slope value (59.92) (Fig. 4(b)). Population density is highest in the Jessore district and moderate in the north eastern part such as Feni and south eastern part of such as the Chittagong and Cox's Bazar (Fig. 4(c)). Population density is comparatively lower 108–1249 in most part of the coastal region. Rural population is comparatively higher south western part of the region such as Shatkhira and Khulna. Moderate rural population dominate the southern region like Bagerhat and some part of the Chittagong district (67.47–84.10) (Fig. 4(d)). Female population percentage is highest in the Feni and Jhalakati district (Fig. 4(e)). Bagerhat and some part of the Chittagong district contains the lowest percentage of female population which is around 44.58–48.01%. Western part of the region such as Khulna, Shatkhira and Bagerhat contains moderate female population percentage (49.77–50.92). South eastern parts of the coastal region such as the Chittagong, Cox's bazar and Feni contains high (18.62–22.65) and moderate population (16.37–18.61) between 0 and 6 years old. Population between 0- and 6-years old percentages is comparatively lower in the western and south western part of the region such as the Bagerhat, Khulna and Shatkhira (Fig. 4(f)). Older population dominate in the districts like Patuakhali, Borgona, Jhalakati etc. Population ages 65 and above (%) is comparatively lower in the Chittagong and Cox's Bazar district (1.66–3.56) (Fig. 4(g)). Poverty head count ratio is higher in the south western part and northern part of the region. Districts like Shatkhira, Chandpur and Borgona contains higher level of poverty head count ratio which is around (46.57–64.37) percent (Fig. 4(h)). Poverty head count ratio is comparatively lower in the districts like Chittagong and Feni (0.01–10.48). Moderate poverty headcount ratio dominates districts like Jessore and Khulna. People living in the districts like Bhola, Noakhali, Shatkhira and Khulna mostly depend on the agriculture (68.53–81.62). Dependency upon agriculture is comparatively low in the districts like Feni, Bagerhat (17.83–38.24) and lowest in some parts of the Chittagong (0.26–117.82 (Fig. 4(i)).

#### 3.1.3 Description of the adaptive capacity parameters

**Fig. 5:**
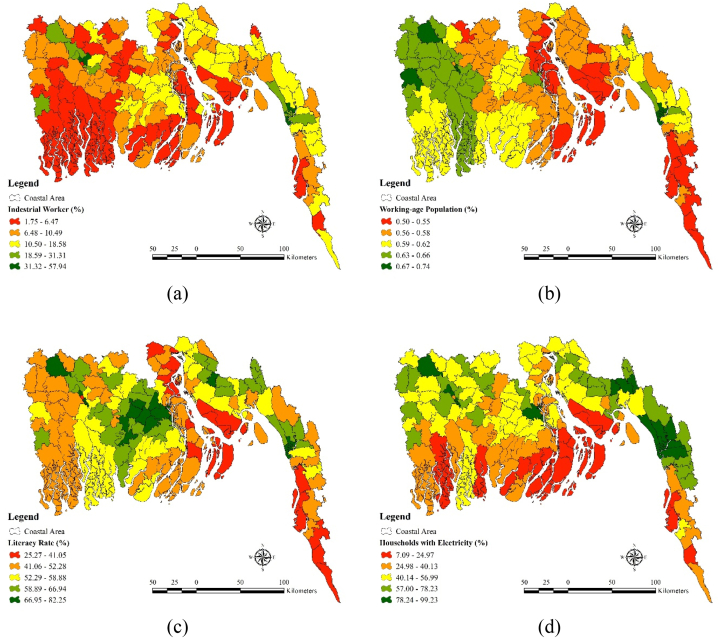

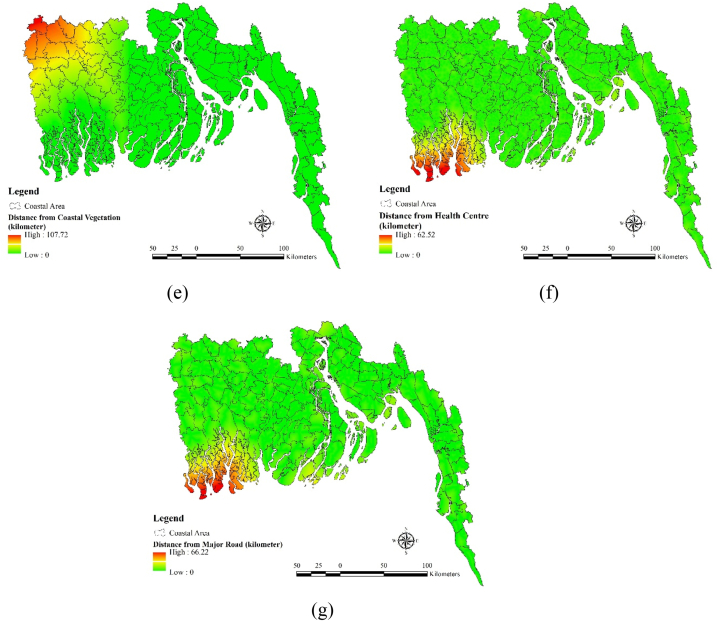
Adaptive Capacity Factors: (a) Industrial worker, (b) Working age population, (c) Literacy rate, (d) Households with electricity, (e) Distance from coastal vegetation, (f) Distance from health center, (g) Distance from major road.

In the case of adaptive capacity seven factors such as i) industrial worker (%), ii) working age population (%) iii) literacy rate iv) households with electricity (%) v) distance from coastal vegetation (kilometer) vi) distance from health center (kilometer) vii) distance from major road (kilometer) have been used as adaptive capacity factors. Industrial worker percentage is comparatively lower (1.75–6.47) in the districts like Khulna, Shatkhira, Bagerhat, Cox's Bazar, Chittagong, Jessore. Moderate industrial worker percentage (10.50–18.58) can be found in the districts like Feni, Jhalokati, Barisal etc (Fig. 5(a)). Working age population percentage is higher in the North western districts like Shatkhira, Khulna and Bagerhat (Fig. 5(b)). On the other hand, districts like Cox's Bazar, Noakhali and Bhlola contains comparatively lower (0.50–0.55) working age population. Literacy rates are higher (66.95–82.25) in the districts like Barisal, Pirojpur, Jhalokati etc (Fig. 5(c)). Districts like Noakhali, parts of Chittagong hill tracks Bhola contains very low (25.27–41.05) level of literacy rate. Household electricity percentage is very scarce (7.09–24.97) in Noakhali, Bagerhat, Bhola, Shariatpur and hilly parts of Cox's Bazar. Electricity percentage is pretty higher in the parts of Chittagong (78.24–99.23) (Fig. 5(d)). Distance from coastal vegetation is pretty close to the most of the districts situated in coastal region (Fig. 5(e)). Only some parts of Jessore and Shatkhira are situated pretty far from the coastal region (Fig. 5(f)). Health center is pretty much available in most of the coastal region districts but due to Sundarbans Mangrove forest health centers are not available in a big portion of Shatkhira and Khulna district. Same type of scenario can be seen in the case of distance from major road (kilometer) (Fig. 5(g)).

### 3.2 Principal component analysis outputs

The data set was appropriate for factor analysis as shown by the positive result of the Bartlett's Test of Sphericity (P 0.05). Sensitivity (0.670), adaptability (0.624), and exposure (0.500) KMO tests all demonstrated substantial information overlap across the variables. To minimize the multicollinearity between different independent variables factor analysis is a viable method. In this study, sensitivity and adaptive capacity were considered for principal component analysis. However, for the exposure variable, no multicollinearity was detected, and instead, normalization was performed to mitigate the issue of varying distances. This approach allowed for a comprehensive and efficient reduction of redundant information, facilitating the analysis of cyclone-related vulnerability within the dataset. Kaiser Eigenvalue criterion and scree plots depicts that 2 component of sensitivity (86.32 %) and (16.67 %) and 2 components of adaptive capacity (54.7 %) and (36.13 %) were identified (Table 3).

**Table 3:** Factor loading of the input variable.

Factors		Components		% of Variance (1)	% of Variance (2)	Cumulative
		1	2			
Sensitivity	Elevation (m)	0.035	0.027	83.321	16.679	90.00
	Slope	−0.023	0.123			
	Population Density (sq. km)	−1	0.80			
	Rural Population (%)	0.806	0.075			
	Female Population (%)	0.575	0.473			
	Population between 0 and 6 years old (%)	0.364	0.236			
	Population ages 65 and above (%)	0.545	0.632			
	Poverty headcount ratio (%)	0.414	0.321			
	Primary employment: Agriculture (%)	0.617	0.213			
Adaptive Capacity	Working-age Population (%)	0.75	0.137	54.70	36.13	90.93
	Primary employment: Industry (%)	0.696	−0.27			
	Literacy Rate (%)	0.755	−0.25			
	Households with Electricity (%)	0.964	−0.23			
	Distance from Coastal Vegetation (km)	0.416	0.909			
	Distance from Health Centre (km)	−0.27	0.002			
	Distance from Major Road (km)	−0.32	0.07			

In the case of sensitivity, 2 component explained (86.32 %) and (16.67 %) of the input variable. Six factors in component 1- rural population (%) (0.806), female population (%) (0.575), population between 0 and 6 years old (%) (0.364), population ages 65 and above (%) (0.414) and finally primary employment: agriculture (%) 0.617 contributed positively and significantly to sensitivity. On the other hand, population density (sq. km) and population ages 65 and above (%) contributed significantly to the component 2 (0.80 and 0.632). Four factors in component 1- primary employment: industry (%) contributed (0.696), working-age population (%) contributed (0.75), literacy rate (%) contributed (0.755) and household with electricity (%) contributed (0.964) to the adaptive capacity. Component 2 isolates distance from coastal vegetation (km) (0.909) as the only positive factor. So, it can be said that factors like distance to capacity has a high impact on cyclone exposure (Table 3), Six factors such as rural population (%), female population (%), population between 0 and 6 years old (%), population ages 65 and above (%) and finally primary employment: Agriculture (%) have high impact on sensitivity. On the other hand, factors like primary employment industry (%), working-age population (%), literacy rate (%) and household with electricity (%) have a significant impact upon cyclone adaptive capacity (Table 3).

### 3.3 Exposure, sensitivity, adaptive capacity and vulnerability

3.3

**Fig. 6:**
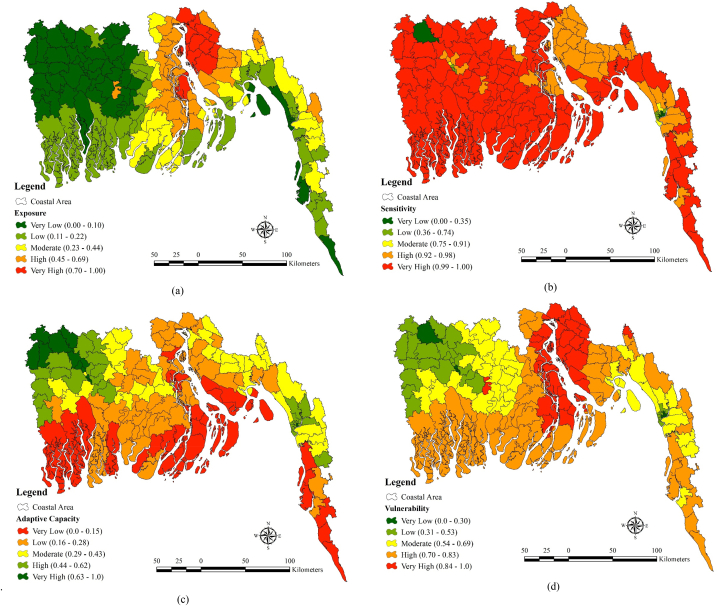
(a) exposure, (b) sensitivity, (c) adaptive capacity, (d) vulnerability.

From the (Fig. 6(a)) it can be said that exposure is very low (0.00–0.10) in the north western region especially in the parts of Jessore, Khulna, Narail, Gopalgonj, Bagerhat and some parts of the Cox's Bazar district. Low exposure can be seen in the parts of the Khulna, Bagerhat and Shatkhira which is mainly the portion of Sundarbans. Very high (0.70–1.00) type of exposure region can be seen in the districts like Chandpur and Lakshmipur. Districts like Barguna and parts of Chittagong as well as Cox's bazar are in the moderate type of exposure zone (0.23–0.44). High type of exposure zone can be seen in the districts like Barisal, Bhola and Noakhali. Sensitivity is very high in almost all the regions and high type of sensitivity can be seen in the districts like Noakhali, Lakshmipur and Chandpur (Fig. 6(b)). Adaptive capacity is very low (0.0–0.15) regions close to the sea such as Cox's Bazar, Shatkhira, Bagerhat, Noakhali, Bhola etc (Fig. 6(c)). On the contrary, very high adaptive capacity (0.63–1.0) can be seen the regions very far from the sea. Low (0.16–0.28) adaptive capacity can be seen in the regions like Jhalokati, Pirojpur and Barguna. Finally, from (Figure) it can be said that Vulnerability is very high (0.84–1) in the regions like Noakhali, Lakshmipur, Chandpur, Bhola. Highly vulnerable zone (0.70–0.83) can be identified in the Mangrove forest which is part of Shatkhira, Bagerhat and Khulna district, Noakhali, parts of Chittagong and Cox's Bazar, Bhola, Patuakhali and Barguna. Low vulnerable zone (0.31–0.53) can be identified in Jessore, Khulna etc. Moderate vulnerable zone dominates in the Barisal, Narail, Gopalgonj and Jhalokati (Fig. 6(d)).

## 4 Discussion

Several studies have been done to detect cyclone vulnerability in national and union level [73]. The majority of the research evaluated exposure to danger on a regional or even a neighborhood scale. But none of the studies have considered important vulnerability factors such as exposure, sensitivity and adaptive capacity as a whole to detect vulnerability in the coastal region of Bangladesh.

First of all, various factors have been identified to use a variable for sensitivity, adaptive capacity and exposure. Factors used for exposure such as slope, elevation, distance to cyclone track, distance to coastline etc has been used as physical vulnerability factors in the study done by [39]. In the same way, factors used in the sensitivity are found to be quite commonly used as social vulnerability in previous study where some factors used in the study as an input variable for adaptive capacity like major roads and distance from coastal vegetation are used as mitigation capacity [39]. But variables like household electricity, industrial worker percentage, working age population etc. haven't been used in the previous studies. Then this study has analyzed whether the variables are applicable for factor analysis. Bartlett's Test of Sphericity (P 0.05) showed that it is quite applicable which is verified by other studies as well [70]. After that different variables used as the input for exposure was analyzed with PCA to see which variables contribute most to the exposure, sensitivity and adaptive capacity. PCA results shows that distance to coastline contributed (0.998) significantly and positively to the cyclone exposure which is also seen in the previous studies [73]. Chandpur and Lakshmipur are in the very high exposure zone. Districts like Barguna and parts of Chittagong as well as Cox's bazar are in the moderate type of exposure zone Barisal, Bhola and Noakhali are the regions with high risk zone. Six factors in component 1- rural population (%) (0.806), female population (%) (0.575), population between 0 and 6 years old (%) (0.364), population ages 65 and above (%) (0.414) and finally primary employment: agriculture (%) 0.617 explained sensitivity. It can be said that influence of rural population percentage is the highest upon sensitivity. Factors like agricultural primary employment and female education also plays an important role to define sensitivity in the region. These three factors have been used as an parameter for social vulnerability where these factors played an important influence to define vulnerability [73].

Four factors in component 1 such as industry (%) contributed (0.696), working-age population (%) contributed (0.75), literacy rate (%) contributed (0.755) and household with electricity (%) contributed (0.964) explained adaptive capacity. It can be said that districts household with higher electricity percentage contains better adaptive capacity than other regions and literacy rate percentage also plays an important role to mitigate cyclone disaster [62]. Cox's Bazar, Shatkhira, Bagerhat, Noakhali, Bhola etc are in the very low adaptive capacity zone where regions like Jessore, Khulna and Gopalgonj are in the very high adaptive capacity zone due to better household electricity percentage and literacy rate. In the case of sensitivity, very high zones can be found in almost all the part of the region except for the districts like Noakhali, Lakshmipur and Chandpur where sensitivity is high.

The analytical framework employed in conjunction with this study's findings. Separating exposure, sensitivity, and adaptive capacity enables a more precise assessment of a cyclone vulnerability of a district [70,73]. For example, some parts of Jessore have higher sensitivity but due to its higher adaptive capacity the vulnerability is low. Using Equations (1) and (2) the vulnerability of the coastal region has been assessed. Findings show that Noakhali, Lakshmipur, Chandpur, Bhola are in the very high vulnerable zone which is pretty similar to the study done by [39,73]. [81] have identified these districts very highly vulnerable for cyclone coastal flooding. [82,83] showed that cyclone occurrence ratio is quite high in these region and causalities are more devasting than other coastal districts of Bangladesh. Moreover, Bhola has been ranked as the highest vulnerable district by [71].On the other hand, districts like Shatkhira, Bagerhat, Khulna, Noakhali, parts of Chittagong and Cox's Bazar, Bhola, Patuakhali and Barguna are situated in the high vulnerable zone. But study done by [73] identified Cox's bazar, Noakhali and areas of Khulna and Shatkhira close to Sundarban as the very high vulnerable zone. This study has also added parts of Bhola, Gopalgonj and Chandpur in the very high susceptible zone. Where study done by [81] have identified Khulna and Cox's high vulnerable zone for cyclone flooding. Moderate type of cyclone vulnerable zone dominates in Barisal, Narail, Gopalgonj and Jhalokati. [71] have ranked Narail as the 6th risk area and ranked Barisal as the 7th risky district among 19 coastal district of Bangladesh. Low susceptible zone areas can be found in Jessore and parts of Khulna far from Sundarbans. Previous studies done by [39,73] have also identified these areas as low vulnerable zone. But [81] have identified whole region of Khulna and Shatkhira highly vulnerable for coastal flooding. A full understanding of the relationship between exposure, sensitivity, and adaptive capacity is crucial for assessing cyclone susceptibility in the coastal region of Bangladesh. These elements are not independent; rather, they are intimately interconnected, and their interplay has a substantial influence on the region's overall susceptibility. For instance, based on our data analysis, it can be inferred that towns such as Chandpur and Lakshmipur are situated in a region characterized by an exceptionally high level of exposure. Their proximity to the coast places them in a physically vulnerable position, so exposing them to heightened risks. The geographical exposure of a region sets the foundation for potential vulnerability, rendering it a crucial factor to be taken into account in the comprehensive evaluation. Rural areas characterized by a significant reliance on agriculture and a greater concentration of vulnerable demographics, such as children and the elderly, exhibit heightened sensitivity to the consequences of cyclones. Furthermore, the impact of female education and the characteristics of primary work are significant factors in shaping the concept of sensitivity. These three characteristics have also been acknowledged as variables for social vulnerability, so underscoring their pivotal significance in delineating the broader concept of vulnerability. The third aspect, known as adaptive capability, pertains to the region's capacity to effectively respond to and recuperate from cyclone events. The interaction among these components is readily apparent. For example, districts with a higher percentage of households having access to electricity exhibit better adaptive capacity. The availability of resources, such as electricity, and a higher level of literacy within a community contribute to the bolstering of resilience in the event of cyclone disasters. This observation underscores the significance of literacy and the availability of resources in reducing the adverse effects of cyclones. The comprehensive evaluation of vulnerability within the coastal zone necessitates the acknowledgment of the intricate interplay among these three components. As an illustration, a region characterized by little exposure but heightened sensitivity may nonetheless have elevated vulnerability if its adaptive capacity proves inadequate in effectively managing the consequences of cyclone events. The recognition of these interconnections highlights the intricate nature of vulnerability within the region.

In this region, the detection of coastal vulnerability, exposure, sensitivity, and adaptive capacity using Principal component analysis and geospatial techniques is relatively novel. Furthermore, it is possible to draw comparisons between our research findings and the experiences seen in neighboring nations situated in the Bay of Bengal region, namely India and Myanmar. These nations have comparable geographical and meteorological characteristics, and they similarly encounter issues associated with cyclones [16,19]. Through the process of drawing parallels and conducting an analysis of regional risks, our study has the potential to provide a valuable reference for neighboring nations who are grappling with similar difficulties. The collective experiences and assessments of individuals can play a significant role in fostering regional collaboration and shaping policies aimed at mitigating the risks associated with cyclone disasters. This would add a new dimension to vulnerability assessments, making it simpler and more efficient to take the necessary steps to mitigate cyclone damage. This would also aid policymakers in determining the variables accountable for cyclone destruction. The most significant limitations are the lack of a physical study and the reliance on secondary sources of data for the majority of the study's analysis. Complex and situationally dependent, the notions of vulnerability, exposure, sensitivity, and adaptation capacity in coastal areas affected by many socioeconomic and environmental variables. Decision-making may be hindered by PCA and GIS approaches since they may not be able to capture the intricacies of these elements. Assumptions about the data and statistical correlations are necessary for PCA and GIS methods to work. A transferable approach for identifying and treating coastal cyclone hazards makes our study relevant internationally. PCA and GIS methods provide a model for vulnerable coastal areas worldwide. The findings further emphasize the global necessity of managing cyclone and natural catastrophe vulnerabilities. Our findings can help international audiences develop data-driven disaster risk reduction strategies, collaborate on research and knowledge sharing, and build more resilient coastal regions and reduce the impact of cyclone disasters on communities and economies worldwide. It is important to acknowledge the limitations of this investigation. One primary limitation is the lack of a physical study, which is essential for a more comprehensive vulnerability assessment. Additionally, the study relied on secondary data sources for a significant portion of its analysis. Vulnerability, sensitivity, exposure, and exposure are all inherently situationally dependent and complex concepts. While the research acknowledges the intricacy of these concepts, employing PCA and GIS methods could potentially oversimplify them. These methodologies might not comprehensively encompass the complexities of human behaviour, social dynamics, and environmental conditions, which could potentially restrict the thoroughness and precision of vulnerability assessments. Inherent in the process of data analysis are assumptions. PCA and GIS methods depend on data-related assumptions and statistical correlations to function effectively. Potential uncertainties may arise during the vulnerability assessment due to these assumptions; any deviations from these assumptions may have an adverse effect on the precision of the outcomes. The complex and situationally dependent nature of vulnerability in coastal areas underscores the need for on-ground data collection and surveys to enhance the accuracy and depth of future research.

## 5 Conclusion

PCA is particularly useful for representing multivariate data tables as summary indices to identify trends, leaps, clusters, and outliers. This summary may reveal observations-variable and variable- connections. In this study PCA along with geospatial technique have been used to detect exposure, sensitivity, adaptive capacity and finally vulnerability. First of all, the socioeconomic and physical variables contribute most to the vulnerability factors have been identified. Then exposure, sensitivity and adaptive capacity maps have been created based on those influencing variables. Finally, vulnerability map has been created using those factors. Findings showed that Noakhali, Lakshmipur, Chandpur, Bhola are in the very high vulnerable zone. On the other hand, districts like Shatkhira, Bagerhat, Khulna, Noakhali, parts of Chittagong and Cox's Bazar, Bhola, Patuakhali and Barguna are situated in the high vulnerable zone. Moderate type of cyclone vulnerable zone dominates in Barisal, Narail, Gopalgonj and Jhalokati. Low susceptible zone areas can be found in Jessore and parts of Khulna far from Sundarbans. Validation of the work using impact dataset of more historical cyclones is suggest by [73] for future scope which has been done in this study by using ROC curve. Since Bangladesh is still a developing nation, its coastal residents do not have access to health or property insurance. The yearly budget does not include any designated funds to compensate for cyclone damage. Assessing the long-term spatial distribution of casualty and livelihood vulnerability of the coastal people to cyclones and not just investing all vulnerable reduction actions in the areas that were most affected by the last cyclone is essential to ensure an efficient use of the rehabilitation funds provided after each disaster by international donors. This study might therefore serve as a baseline document for local and national disaster management to adopt and effectively execute structural and non-structural disaster vulnerability reduction measures both before and after the catastrophe. It would help policy makers, environmental analyst, planners and government officials to take on necessary steps for each district based on their level of risk or vulnerability. As we move forward, it is critical to consider several avenues for future research. One valuable direction is the prediction of cyclone vulnerability for upcoming years, considering the dynamic nature of climatic conditions. Additionally, exploring vulnerability in the context of Representative Concentration Pathways (RCP) is essential, as it aligns with the global goal of climate change adaptation.

## CRediT authorship contribution statement

**Showmitra Kumar Sarkar:** Writing – review & editing, Writing – original draft, Visualization, Validation, Supervision, Software, Resources, Project administration, Methodology, Investigation, Funding acquisition, Formal analysis, Data curation, Conceptualization. **Rhyme Rubayet Rudra:** Writing – review & editing, Writing – original draft, Visualization, Validation, Software, Resources, Methodology, Investigation, Formal analysis, Data curation. **Md. Mehedi Hasan Santo:** Writing – original draft, Visualization, Validation, Software, Investigation, Data curation.

## Declaration of competing interest

The authors declare that they have no known competing financial interests or personal relationships that could have appeared to influence the work reported in this paper.

## References

[1] Hoque M.A., Phinn S., Childs I. (2017). Tropical cyclone disaster management using remote sensing and spatial analysis: a review. Int. J. Disaster Risk Reduct..

[2] Hoque M.A., Pradhan B., Ahmed N., Roy S. (2019). Tropical cyclone risk assessment using geospatial techniques for the eastern coastal region of Bangladesh. Sci. Total Environ..

[3] Chowdhury H., Scholar G. (2023).

[4] Li K., Sheng G. (2013).

[5] Weinkle Jessica R.M. (2012).

[6] Murakami H., Wang B., Li T., Kitoh A. (2013). Projected increase in tropical cyclones near Hawaii. Nat. Clim. Chang..

[7] Chowdhury H., Scholar G. (2023).

[8] Mendelsohn R. (2011).

[9] Varotsos C.A., Efstathiou M.N. (2013). Is there any long-term memory effect in the tropical cyclones ?.

[10] Roy P., Agricultural N.C. (2020).

[11] Alam E., Dominey-howes D. (2015).

[12] Walsh K.J.E., Camargo S.J., Knutson T.R., Kossin J., Lee T.-C., Murakami H., Patricola C. (2018).

[13] Glenn E.P., Hodges C.N., Lieth H., Pielke R., Pitelka L. (2015). Climate Change 2014.

[14] Rahman M.M., Hossain M.A., Ali M.R., Ahmed Z., Hedayutul Islam A.H.M. (2022). Assessing vulnerability and adaptation strategy of the cyclone affected coastal area of Bangladesh. Geoenvironmental Disasters.

[15] Md P.R., Ahmed Ashik, Paul Argho Debobrata (2021). Recycling of cotton dust for organic farming is a pivotal replacement of chemical fertilizers by composting and its quality analysis.

[16] Saravanan S., Abijith D., Kulithalai Shiyam Sundar P., Reddy N.M., Almohamad H., Al Dughairi A.A., Al-Mutiry M., Abdo H.G. (2023). Multi-criterion analysis of cyclone risk along the coast of Tamil Nadu, India—a geospatial approach. ISPRS Int. J. Geo-Information..

[17] Boragapu R., Guhathakurta P., Sreejith O.P. (2023). Tropical cyclone vulnerability assessment for India. Nat. Hazards.

[18] Prasanta Patri S.K.P., Sharma Pritee (2022). A multidimensional model for cyclone vulnerability assessment of urban slum dwellers in India: a case study of Bhubaneswar city, International Journal of Disaster Risk Reduction. Int. J. Disaster Risk Reduct..

[19] Saravanan S., Jennifer J., Singh L., Abijith D. (2018). Cyclone vulnerability assessment of cuddalore coast in Tamil Nadu, India using remote sensing, and GIS. MATEC Web Conf.

[20] Ghosh S., Mistri B. (2023). Cyclone-induced coastal vulnerability, livelihood challenges and mitigation measures of Matla–Bidya inter-estuarine area, Indian Sundarban. Nat. Hazards.

[21] Das M., Das A., Momin S., Pandey R. (2020). Mapping the effect of climate change on community livelihood vulnerability in the riparian region of Gangatic Plain, India. Ecol. Indic..

[22] Nazeer M., Waqas M., Shahzad M.I., Zia I., Wu W. (2020). Coastline vulnerability assessment through landsat and cubesats in a coastal mega city. Rem. Sens..

[23] Salik K.M., Jahangir S., Zahdi W. ul Z., ul Hasson S. (2015). Climate change vulnerability and adaptation options for the coastal communities of Pakistan. Ocean Coast Manag..

[24] Hamidi A.R., Zeng Z., Khan M.A. (2020). Household vulnerability to floods and cyclones in Khyber Pakhtunkhwa, Pakistan. Int. J. Disaster Risk Reduct..

[25] Rafiq L., Blaschke T. (2012). Disaster risk and vulnerability in Pakistan at a district level, Geomatics, Nat. Hazards Risk.

[26] Neckel J., Niklas Kropáček (2018).

[27] Bhandari D., Neupane S., Hayes P., Regmi B., Marker P. (2020).

[28] Cortés-Ramos J., Farfán L.M., Herrera-Cervantes H. (2020). Assessment of tropical cyclone damage on dry forests using multispectral remote sensing: the case of Baja California Sur, Mexico. J. Arid Environ..

[29] Deng Z., Xun H., Zhou M., Jiang B., Wang S., Guo Q., Wang W., Kang R., Wang X., Marley G., Ma W. (2015). Impacts of tropical cyclones and accompanying precipitation on infectious diarrhea in cyclone landing areas of Zhejiang Province, China. Int. J. Environ. Res. Publ. Health.

[30] Alam A., Sammonds P., Ahmed B. (2019).

[31] Sattar A., Cheung K.K.W. (2019). International Journal of Disaster Risk Reduction Tropical cyclone risk perception and risk reduction analysis for coastal Bangladesh : household and expert perspectives. Int. J. Disaster Risk Reduct..

[32] UNDRR (2009).

[33] Hoque M.A., Phinn S., Roelfsema C., Childs I., Hoque M.A., Phinn S., Roelfsema C. (2016). Assessing tropical cyclone impacts using object- based moderate spatial resolution image analysis : a case study in Bangladesh moderate spatial resolution image analysis : a case study. Int. J. Rem. Sens..

[34] Chowdhury H., Asiabanpour B. (2023).

[35] Ali Sa A.S., Khatun R., Ahmad A. (2020).

[36] Mazumdar J., Paul S.K. (2017). Author ’ s Accepted Manuscript cyclones A spatially explicit method for identification of vulnerable hotspots of Odisha , India from potential cyclones. Int. J. Disaster Risk Reduc..

[37] Phinn S., Roelfsema C., Childs I.R. (2016).

[38] Chowdhury H. (2020).

[39] Hoque M.A., Pradhan B., Ahmed N., Alamri A.M. (2021). Cyclone vulnerability assessment of the western coast of Bangladesh Cyclone vulnerability assessment of the western coast. Geomatics, Nat. Hazards Risk.

[40] Ajim S., Rumana A., Ateeque K., Syed A., Ahmad N. (2019). Assessment of cyclone vulnerability , hazard evaluation and mitigation capacity for analyzing cyclone risk using GIS technique : a study on sundarban biosphere reserve , India. Earth Syst. Environ..

[41] Yves Hategekimana F.L., Lijun Yu Yueping Nie, Zhu Jianfeng (2018). Integration of multi-parametric fuzzy analytic hierarchy process and GIS along the UNESCO World Heritage. Nat. Hazards.

[42] C. Wisner, P.I. Framework, at Risk : Natural Hazards , People ’ S Vulnerability and Disasters Second Edition the Attached Three Chapters Constitute Part I of the Book , and Have Been Made Available in the Public Domain by the Authors and Routledge as Part of the UNDP Follow up to the Hyogo Framework for Action 2005. Royalties for the Print Versions of the Book Are Donated to Three Disaster Reduction Networks in the South : La Red (Latin America), Duryog Nivaran (South Asia) and Peri-Peri (Southern Africa) Contents, (n.d.).

[43] Cutter S.L., Cutter S.L. (2013).

[44] Ii B.L.T., Kasperson R.E., Matson P.A., Mccarthy J.J., Corell R.W., Christensen L., Eckley N., Kasperson J.X., Luers A., Martello M.L., Polsky C., Pulsipher A., Schiller A. (2003). A framework for vulnerability analysis in sustainability science.

[45] Brata D., Argha P., Model R. (2023).

[46] Chowdhury H., Scholar G. (2023).

[47] Roy P., Agricultural N.C., Kumer A. (2019).

[48] Sarkar S.K., Rudra R.R., Nur S., Das P.C. (2023). Partial least-squares regression for soil salinity mapping in Bangladesh. Ecol. Indic..

[49] Sarkar S.K., Rudra R.R., Sohan A.R., Das P.C., Ekram K.M.M., Talukdar S., Rahman A., Alam E., Islam M.K., Islam A.R.M.T. (2023). Coupling of machine learning and remote sensing for soil salinity mapping in coastal area of Bangladesh. Sci. Rep..

[50] Haque M.N., Mahi M.M., Sharif M.S., Rudra R.R., Sharifi A. (2023). Changes in the economic value of ecosystem services in rapidly growing urban areas: the case of Dhaka, Bangladesh. Environ. Sci. Pollut. Res..

[51] Mahi M.M., Sharif M.S., Rudra R.R., Haque M.N. (2021). The geo-spatial approach to detect the change in vegetation and land surface temperature (lst) after formation of Rohingya settlements in Bangladesh. J. Civ. Eng. Sci. Technol..

[52] Mahi M.M., Sharif S., Rudra R.R. (2022). Passenger travel behavior before & during the COVID-19 outbreak. A COMPARATIVE ANALYSIS.

[53] Khan T.A., Brata D., Argha P., Anita M.S. (2021).

[54] Haque M.N., Sharif M.S., Rudra R.R., Mahi M.M., Uddin M.J., Ellah R.G.A. (2022). Analyzing the spatio-temporal directions of air pollutants for the initial wave of Covid-19 epidemic over Bangladesh: application of satellite imageries and Google Earth Engine. Remote Sens. Appl. Soc. Environ..

[55] Mondal S., Rudra R.R. (2022).

[56] Rudra R.R., Sarkar S.K. (2023). Artificial neural network for flood susceptibility mapping in Bangladesh Heliyon Artificial neural network for flood susceptibility mapping in Bangladesh. Heliyon.

[57] Brammer H. (2014). Climate Risk Management Bangladesh ’ s dynamic coastal regions and sea-level rise. Clim. Risk Manag..

[58] Ahmad H. (2019).

[59] Iftekhar M.S. (2006). Conservation and management of the Bangladesh coastal ecosystem : Overview of an integrated approach.

[60] Adams H., Neil Adger W., Ahmed M., Huq H., Rahman R., Salehin M. (2018).

[61] Uddin J., Li Y., Cheung K.K., Nasrin Z.M. (2016).

[62] Ahmed B., Kelman I., Fehr H.K., Saha M. (2016).

[63] Hossain N. (2015). International Journal of Disaster Risk Reduction Analysis of human vulnerability to cyclones and storm surges based on in fl uencing physical and socioeconomic factors : evidences from coastal. Int. J. Disaster Risk Reduct..

[64] Fazlul M., Mimura N. (2008).

[65] Mallick B., Ahmed B., Vogt J. (2017).

[66] Shahriar M., Hossain S., Saha D. (2022).

[67] Uddin N., Islam A.K.M.S., Bala S.K., Islam G.M.T., Adhikary S., Saha D., Haque S., Fahad G.R., Akter R. (2019). Mapping of climate vulnerability of the coastal region of Bangladesh using principal component analysis. Appl. Geogr..

[68] Okey T.A., Agbayani S., Alidina H.M. (2015). Ocean & Coastal Management Mapping ecological vulnerability to recent climate change in Canada ’ s Paci fi c marine ecosystems. Ocean Coast Manag..

[69] Thomas A.M.B., Wratten S.D., Sotherton N.W., Wratten S.D. (1992). Predator densities and emigration creation of ’ island ’ habitats in farmland to manipulate populations of beneficial arthropods. PREDATOR DENSITIES AND EMIGRATION.

[70] Sarkar S.K., Morshed M., Chakraborty T. (2022).

[71] Mohammad D., Haque E., Mimi A., Krishna R., Salman A.M. (2020). International Journal of Disaster Risk Reduction Evaluation of natural hazard risk for coastal districts of Bangladesh using the INFORM approach. Int. J. Disaster Risk Reduct..

[72] Hasnat S., Showmitra A., Sarkar K., Ali A., Rina B., Swapan K. (2023). Assessment of coastal vulnerability using integrated fuzzy analytical hierarchy process and geospatial technology for effective coastal management. Environ. Sci. Pollut. Res..

[73] Quader M.A. (2017).

[74] Brooks N. (2017).

[75] Panel T.I., Change C., Nations U., Programme T E., Ipcc, Report F.A., Report T., Change C. (2007).

[76] Tan B., Uk V.M., Uk M.P., Germany J.P. (2012).

[77] Baeza C., Lantada N., Amorim S. (2016).

[78] Ghosh S., Saha S., Bera B. (2022). Flood susceptibility zonation using advanced ensemble machine learning models within Himalayan foreland basin. Nat. Hazards Res..

[79] Mahmoudi M.R., Heydari M.H., Qasem S.N., Mosavi A., Band S.S. (2020). Principal component analysis to study the relations between the spread rates of COVID-19 in high risks countries principal component analysis to study the relations between the spread rates of COVID-19 in high risks countries. Alexandria Eng. J..

[80] Mr Brett Williams T.B., Onsman Andrys (2010). Exploratory factor analysis: a five-step guide for novices.

[81] Bernard A., Long N., Becker M., Khan J., Fanchette S., Rochelle L., Cnrs U., De Gouges O., Rochelle L. (2022).

[82] Ahamed S. (2012). Reducing cyclone impacts in the coastal areas of Bangladesh. A Case Study of Kalapara Upazila.

[83] Shamrat M.R.I. (2018).

